# Supplementary material for: An investigation of perceptual biases in complex regional pain syndrome
Source: PeerJ. 2020 Apr 2;8:e8819. doi: 10.7717/peerj.8819 (PMC7130113; doi:10.7717/peerj.8819)
Supplement: Supplemental Information 1 [file peerj-08-8819-s001.doc]

**Appendix**

1. **Diagnostic screening**

In order to confirm the diagnosis of CRPS in the CRPS group and to exclude CRPS in the wrist and shoulder pain groups, a diagnostic screening procedure was performed on all participants. The procedure was based on the Budapest criteria for the research diagnosis of CRPS [1,2]. Participants received the research diagnosis of CRPS when i) they experienced continuing pain, which was disproportionate to any inciting event; ii) they reported at least 1 symptom in each of four symptom categories (sensory, vasomotor, sudomotor/edema, motor/trophic); iii) they displayed minimum 1 symptom in at least two of four sign categories (sensory, vasomotor, sudomotor/edema, motor/trophic); and iv) there was no other diagnosis that better explained the signs and symptoms. The first and last criterion were evaluated by the responsible doctor, the second and third were assessed by the experimenter by, respectively, interviewing and testing the participants.

### Self-reported symptoms

Participants were asked by the experimenter which of the following symptoms they experienced from the four symptom categories: continuing pain, hyperesthesia and/or allodynia (sensory category), temperature asymmetry and/or skin color changes and/or skin color asymmetry (vasomotor category), edema and/or sweating changes and/or sweating asymmetry (sudomotor/edema category), and decreased range of motion and/or motor dysfunction (weakness, tremor, dystonia) and/or trophic changes (hair, nails, skin) (motor/trophic category).

### Signs observed on examination

The experimenter assessed which of the following symptoms from the four sign categories were present at the time of the experiment: hyperalgesia (to pinprick) and/or allodynia (to heat/cold and to brush stroking) (sensory category), temperature asymmetry and/or skin color changes and/or skin color asymmetry (vasomotor category), edema and/or sweating changes and/or sweating asymmetry (sudomotor/edema category), and decreased range of motion and/or motor dysfunction (weakness, tremor, dystonia) and/or trophic changes (hair, nails, skin) (motor/trophic category).

### Sensory symptoms. Hyperalgesia was assessed by subsequently pricking the non-painful and the painful limb twice with a Semmes-Weinstein monofilament no. 19 (SENSELab Aesthesiometer, Hörby, Sweden). Participants reported pain intensity both times on a Likert scale ranging from 0 (“no pain”) to 10 (“worst imaginable pain”). The symptom was evaluated as positive when the maximal pain rating of the painful limb minus the maximal pain rating of the non-painful limb ≥ 3. Thermal allodynia was measured by subsequently rolling a cold (25°C) and a warm (40°C) metal roller (SENSELabTM, Rolltemp, Hörby, Sweden) twice over the non-painful and the painful limb (SenseLab, n.d.-a). The experimenter also stroked (~2s, ~5cm distance) these locations twice with a small brush (~22mm wide) to measure brush allodynia (SenseLab, n.d.-b). Pain intensity was reported twice for each hand on a Likert scale ranging from 0 (“no pain”) to 10 (“worst imaginable pain”). Thermal and brush allodynia were considered present when the maximal pain rating of the painful limb minus the maximal pain rating of the non-painful limb ≥ 3.

### Vasomotor symptoms. An infrared thermometer (Hartmann, Thermoval® duo scan, Heidenheim, Germany) was used to measure the temperature of the painful and the non-painful limb. Based on Perez [3], temperature asymmetry was defined as a difference of 0.4°C or more between both limbs (in either direction). Changes and/or asymmetry in skin color were assessed by observing the painful and non-painful limb.

### Sudomotor symptoms/edema. The circumference of both hands and wrists was measured with a flexible measuring tape (SECA). Edema was defined as a minimal difference in circumference of 6.5% between the painful and the non-painful limb [3]. Sweating changes and/or asymmetry were assessed by observing and touching the skin of the painful and non-painful limb.

### Motor/trophic symptoms. Dorsal and palmar flexion (degrees in °) of the wrist was measured with an inclinometer (BASELINE®) and added up to calculate the active range of motion (AROM). A decreased AROM was present when the AROM of the painful limb was more than 15% smaller than the AROM of the non-painful limb [3]. Motor dysfunctions (tremor, weakness and dystonia) and trophic changes of the nails, hair and skin were assessed through observation.

Table A1. Number of fulfilled criteria per symptom category and per sign category according to the Budapest criteria for all participants

| **Reported symptoms** | | | | | | | **Signs** | | | |
| --- | --- | --- | --- | --- | --- | --- | --- | --- | --- | --- |
|  | **ID** | **Sensory** | **Vasomotor** | **Sudomotor/Edema** | **Motor/Trophic** | **Sensory** | | **Vasomotor** | **Sudomotor/Edema** | **Motor/Trophic** |
| **CRPS** | 1 | 2/2 | 1/2 color | 1/2 E | 3/3 | 0/3 | | 1/2 color | 0/2 | 3/3 |
|  | 2 | 2/2 | 2/2 | 1/2 S | 2/3 range+motor | 2/3 H+TA | | 0/2 | 0/2 | 3/3 |
|  | 3 | 2/2 | 2/2 | 1/2 E | 2/3 range+motor | 1/3 H | | 0/2 | 0/2 | 2/3 range+motor |
|  | 4 | 2/2 | 2/2 | 1/2 E | 3/3 | 2/3 H+TA | | 1/2 color | 0/2 | 3/3 |
|  | 5 | 2/2 | 1/2 color | 1/2 E | 2/3 range+motor | 2/3 H+TA | | 0/2 | 0/2 | 2/3 range+motor |
|  | 6 | 1/2 H | 1/2 t° | 1/2 E | 3/3 | 0/3 | | 2/2 | 0/2 | 3/3 |
|  | 7 | 1/2 A | 2/2 | 1/2 E | 2/3 range+motor | 2/3 H+TA | | 0/2 | 0/2 | 2/3 range+motor |
|  | 8 | 2/2 | 2/2 | 1/2 E | 3/3 | 3/3 | | 1/2 color | 0/2 | 3/3 |
|  | 9 | 1/2 A | 2/2 | 1/2 E | 3/3 | 2/3 H+TA | | 2/2 | 0/2 | 2/3 motor+trophic |
|  | 10 | 2/2 | 1/2 color | 1/2 E | 3/3 | 0/3 | | 0/2 | 0/2 | 2/3 range+troph |
|  | 11 | 1/2 A | 2/2 | 2/2 | 3/3 | 0/3 | | 2/2 | 2/2 | 3/3 |
| **Shoulder** | 1 | 0/2 | 0/2 | 0/2 | 1/3 motor | 0/3 | | 1/2 t° | 0/2 | 0/3 |
|  | 2 | 0/2 | 1/2 t° | 0/2 | 2/3 range+motor | 0/3 | | 0/2 | 0/2 | 0/3 |
|  | 3 | 2/2 | 0/2 | 0/2 | 2/3 range+motor | 0/3 | | 0/2 | 0/2 | 0/3 |
|  | 4 | 1/2 H | 0/2 | 0/2 | 1/3 motor | 0/3 | | 0/2 | 0/2 | 1/3 range |
|  | 5 | 1/2 H | 0/2 | 0/2 | 1/3 range | 0/3 | | 0/2 | 0/2 | 1/3 motor |
|  | 6 | 1/2 H | 0/2 | 1/2 E | 2/3 range+motor | 0/3 | | 0/2 | 0/2 | 2/3 range+motor |
|  | 7 | 0/2 | 1/2 t° | 0/2 | 1/3 range | 0/3 | | 0/2 | 0/2 | 0/3 |
|  | 8 | 0/2 | 0/2 | 0/2 | 2/3 range+motor | 0/3 | | 1/2 t° | 0/2 | 0/3 |
|  | 9 | 1/2 H | 2/2 | 1/2 E | 2/3 range+motor | 0/3 | | 0/2 | 0/2 | 1/3 motor |
|  | 10 | 0/2 | 0/2 | 0/2 | 1/3 range | 0/3 | | 0/2 | 0/2 | 0/3 |
|  | 11 | 0/2 | 0/2 | 0/2 | 2/3 range+motor | 0/3 | | 0/2 | 0/2 | 0/3 |
|  | 12 | 0/2 | 1/2 t° | 0/2 | 2/3 range+motor | 1/3 H | | 0/2 | 1/2 E | 1/3 motor |
|  | 13 | 0/2 | 0/2 | 0/2 | 2/3 range+motor | 0/3 | | 0/2 | 0/2 | 0/3 |
|  | 14 | 0/2 | 0/2 | 0/2 | 2/3 range+motor | 0/3 | | 0/2 | 0/2 | 0/3 |
|  | 15 | 0/2 | 0/2 | 0/2 | 2/3 range+motor | 0/3 | | 0/2 | 0/2 | 0/3 |
| **Wrist** | 1 | 2/2 | 2/2 | 2/2 | 3/3 | 0/3 | | 0/2 | 0/2 | 0/3 |
|  | 2 | 2/2 | 1/2 color | 1/2 E | 3/3 | 0/3 | | 1/2 t° | 0/2 | 2/3 range+troph |
|  | 3 | 2/2 | 2/2 | 1/2 S | 2/3 range+motor | 0/3 | | 0/2 | 0/2 | 0/3 |
|  | 4 | 2/2 | 2/2 | 1/2 E | 2/3 range+motor | 0/3 | | 0/2 | 0/2 | 0/3 |
|  | 5 | 2/2 | 1/2 t° | 1/2 E | 1/3 motor | 0/3 | | 0/2 | 0/2 | 1/3 range |
|  | 6 | 2/2 | 2/2 | 1/2 E | 3/3 | 0/3 | | 0/2 | 0/2 | 1/3 trophic |
|  | 7 | 2/2 | 1/2 color | 1/2 E | 2/3 range+motor | 1/3 TA | | 1/2 t° | 0/2 | 2/3 range+motor |
|  | 8 | 0/2 | 1/2 t° | 0/2 | 3/3 | 0/3 | | 0/2 | 0/2 | 1/3 range |
|  | 9 | 1/2 H | 1/2 t° | 1/2 E | 2/3 range+motor | 0/3 | | 0/2 | 0/2 | 1/3 range |
|  | 10 | 2/2 | 2/2 | 1/2 E | 2/3 range+motor | 2/3 BA+TA | | 0/2 | 0/2 | 1/3 range |
|  | 11 | 1/2 A | 2/2 | 1/2 E | 2/3 range+motor | 0/3 | | 0/2 | 0/2 | 0/3 |
|  | 12 | 2/2 | 2/2 | 1/2 E | 3/3 | 0/3 | | 0/2 | 0/2 | 3/3 |
|  | 13 | 0/2 | 2/2 | 1/2 E | 3/3 | 0/3 | | 0/2 | 0/2 | 2/3 range+troph |
|  | 14 | 2/2 | 0/2 | 1/2 E | 3/3 | 3/3 | | 0/2 | 0/2 | 3/3 |
|  | 15 | 0/2 | 2/2 | 1/2 E | 2/3 range+motor | 0/3 | | 0/2 | 0/2 | 2/3 range+motor |
|  | 16 | 0/2 | 0/2 | 0/2 | 2/3 range+motor | 0/3 | | 0/2 | 0/2 | 0/3 |

Note. T°=temperature asymmetry; color=skin color changes; E=edema; range=decreased range of motion; troph=trophic changes; motor=motor dysfunction; H=hyperalgesia; A=allodynia; BA=brush allodynia; TA=thermal allodynia

Table A2. Additional clinical measures

|  | **ID** | **Pain before** | **Pain during** | **T° affected (°C)** | **T° unaffected (°C)** |
| --- | --- | --- | --- | --- | --- |
| **CRPS** | 1 | 2 | 3 | 36.2 | 35.7 |
|  | 2 | 7 | 7 | 36.5 | 36.6 |
|  | 3 | 8 | 8 | 37 | 36.6 |
|  | 4 | 8 | 8 | 36.7 | 36.6 |
|  | 5 | 6 | 0 | 36.1 | 36.5 |
|  | 6 | 0 | 0 | 35.5 | 35.4 |
|  | 7 | 6 | 6 | 37.1 | 36.9 |
|  | 8 | 7 | 7 | 37.1 | 37.2 |
|  | 9 | 6 | 2 | 35.4 | 36 |
|  | 10 | 4 | 4 | n/a | n/a |
|  | 11 | 4 | 2 | n/a | n/a |
| **Shoulder** | 1 | 7 | 0 | 36.6 | 36.6 |
|  | 2 | 6 | 3 | 36.2 | 36.0 |
|  | 3 | 6 | 4 | 36 | 35.8 |
|  | 4 | 0 | 0 | 37.1 | 37.1 |
|  | 5 | 0 | 0 | 34.9 | 34.9 |
|  | 6 | 8 | 1 | 35.8 | 35.8 |
|  | 7 | 2 | 1 | 36.7 | 36.6 |
|  | 8 | 2 | 2 | 36.5 | 36.2 |
|  | 9 | 5 | 0 | 36.6 | 36.7 |
|  | 10 | 3 | 0 | 35.6 | 35.6 |
|  | 11 | 6 | 0 | 36.1 | 36 |
|  | 12 | 6 | 1 | 35.7 | 35.7 |
|  | 13 | 4 | 6 | 35.8 | 35.7 |
|  | 14 | 8 | 4 | 36.4 | 36.3 |
|  | 15 | 5 | 3 | 36.0 | 36.1 |
| **Wrist** | 1 | 4 | 1 | 35.6 | 35.5 |
|  | 2 | 2 | 0 | 35.6 | 35.4 |
|  | 3 | 5 | 0 | 35.9 | 35.8 |
|  | 4 | 1 | 1 | 36.5 | 36.7 |
|  | 5 | 5 | 0 | 36.6 | 36.5 |
|  | 6 | 3 | 1 | 36.8 | 36.8 |
|  | 7 | n/a | 6 | 35.4 | 36.0 |
|  | 8 | 3 | 3 | 36.5 | 36.7 |
|  | 9 | 0 | 1 | 36.6 | 36.5 |
|  | 10 | 5 | 4 | 36.8 | 36.8 |
|  | 11 | 0 | 0 | 35.4 | 36.0 |
|  | 12 | 8 | 4 | 36.5 | 36.5 |
|  | 13 | 2 | 2 | 36.2 | 36.3 |
|  | 14 | 7 | 9 | 36.8 | 36.7 |
|  | 15 | 8 | 7 | 35.9 | 35.6 |
|  | 16 | 6 | 0 | 35.3 | 35.0 |

*Note. Pain before = pain reported just before starting the experiment rated on a 0-10 NRS; Pain during = average pain reported after each experimental block; T° affected = temperature of the affected hand; T° unaffected = temperature of the unaffected hand*

**References**

[1] Harden RN, Bruehl S, Perez RSGM, Birklein F, Marinus J, Maihofner C, Lubenow T, Buvanendran A, MacKey S, Graciosa J, Mogilevski M, Ramsden C, Chont M, Vatine JJ. Validation of proposed diagnostic criteria (the “budapest Criteria”) for Complex Regional Pain Syndrome. Pain 2010;150:268–274.

[2] Harden RN, Bruehl S, Perez RSGM, Birklein F, Marinus J, Maihofner C, Lubenow T, Buvanendran A, MacKey S, Graciosa J, Mogilevski M, Ramsden C, Schlereth T, Chont M, Vatine JJ. Development of a severity score for CRPS. Pain 2010;151:870–876. doi:10.1016/j.pain.2010.09.031.

[3] Perez RSGM, Keijzer C, Bezemer PD, Zuurmond WWA, De Lange JJ. Predictive value of symptom level measurements for complex regional pain syndrome type I. Eur. J. Pain 2005;9:49–56.
